# Prognostic Value of Quantitative Flow Ratio Combined with SYNTAX Scores I/II in Multivessel Coronary Artery Disease: A Small-Sample, Single-Center Study

**DOI:** 10.31083/j.rcm2509329

**Published:** 2024-09-18

**Authors:** Shuyi Zhang, Ming Wang, Qian Gan, Xinrong Zhai, Yang Chen, Shaofeng Guan, Xinxin Xu, Jiasheng Wen, Xinkai Qu, Wenzheng Han

**Affiliations:** ^1^Department of Cardiology, Huadong Hospital Affiliated to Fudan University, 200040 Shanghai, China; ^2^Department of Cardiology, Kunshan Hospital of Traditional Chinese Medicine, 215300 Kunshan, Jiangsu, China

**Keywords:** multivessel disease, SYNTAX score I, SYNTAX score II, quantitative flow ratio, functional SYNTAX score

## Abstract

**Background:**

A fractional flow reserve (FFR)-fixed-SYNTAX score could decrease the number of high-risk patients. This study explored the prognostic value of non-invasive quantitative flow ratio (QFR)-fixed-SYNTAX I/II scores in multivessel disease patients.

**Methods:**

This was a single-center, small-sample, observational study. Multivessel coronary disease patients were enrolled and finished a 1-year follow-up. SYNTAX scores I/II and functional SYNTAX scores I/II based on QFR (cut-off value of 0.85) were calculated for all patients. The composite occurrence of cardiac deaths, any myocardial infarction, or ischemia-driven revascularization were analyzed using a different score system.

**Results:**

A total of 160 patients were stratified into risk groups based on a different scoring system. FSS (functional SYNTAX score) and FSSII (functional SYNTAX score II) reduce the radio of high-risk major adverse cardiovascular events (MACEs), transforming the patients from high-risk to medium- and low-risk. Furthermore, FSSII (hazard ratio (HR): 1.069, 95% CI: 1.025–1.115, *p =* 0.002) showed a better relationship with MACEs than the other score systems. After recalculating SSII, the survival-free ratio stratified by FSSII decreased from 38.46% to 27.27% in the high-risk group and increased from 84.09% to 86.05% in the low-risk group.

**Conclusions:**

FSS or FSSII could decrease the number of high-risk patients compared to SYNTAX score (SS) and FSS. SYNTAX II score (SSII) and FSSII showed a better predictive ability than other scoring systems for under-risk stratification.

## 1. Introduction

The prognosis of patients with complex coronary heart disease (CAD) depends on anatomical complexity, patients’ clinical characteristics, and comorbidities. It has been shown that the clinical outcome of patients with incomplete revascularization (IR) after percutaneous coronary intervention (PCI) is worse than that of patients with complete revascularization (CR) [1, 2]. However, in most previous studies, the completeness of revascularization was judged based on the severity of anatomical stenosis without considering the functional significance of the remaining stenosis [3, 4].

The SYNTAX score (SS) is a comprehensive angiographic scoring system based on coronary artery anatomy and lesion characteristics [5]. However, with the advancement of coronary intervention technologies and coronary stents, purely anatomical SSs can no longer objectively predict the prognosis or outcome of patients. Therefore, based on SS, some scholars calculated patients’ clinical factors, including age, comorbidities, and other factors, and applied them to the SS, defined as the SYNTAX II score (SSII) [2, 6, 7].

However, even though the SSII can effectively reduce data deviations in SYNTAX studies, with increased research on coronary artery hemodynamics and pathophysiology, coronary angiography alone can no longer meet the clinical requirements for the anatomical characteristics or physiological functions of stenotic lesions [8]. The main reason for the need for additional assessments is that coronary angiography cannot be used to accurately evaluate the relationship between stenosis and myocardial ischemia. Many studies have shown that the prognosis of patients with coronary heart disease mainly depends on the presence or absence of myocardial ischemia rather than the degree of coronary stenosis. Interventions for stenosis without functional significance will not benefit patients [9, 10]. Therefore, functional disease is even more important in treating multivessel or diffuse segmental lesions.

Several studies have shown that through SS combined with fractional flow reserve (FFR), the prognosis of patients can be effectively predicted [1, 11, 12]. Quantitative flow ratio (QFR) is a novel method for deriving FFR without the use of pressure wires or the induction of hyperemia [13, 14]. The diagnostic ability of SS combined with QFR in the prognosis of patients with multivessel disease has also been confirmed [15, 16]. In this study, we aimed to test the diagnostic value of these scores by calculating the preoperative and postoperative SSs and those corrected based on QFR in combination with the incidence of major adverse cardiovascular events (MACEs) among patients after 1 year. In addition, we used the final 1-year incidence of MACEs in patients to verify the correct rate of risk stratification and patients’ outcomes.

## 2. Method

### 2.1 Study Population

We enrolled 187 multivessel disease patients who underwent complete revascularization in the Department of Cardiology, Huadong Hospital, from January 2019 to May 2020; a total of 160 patients completed the follow-up.

### 2.2 Scoring Systems

SYNTAX I/II scores and residual SYNTAX I/II scores were calculated based on the official website of SYNTAX (https://syntaxscore.org/). Functional SYNTAX I/II and residual SYNTAX I/II scores were recalculated based on the QFR cut-off value. All lesions recalculated by QFR >0.85 were excluded from the basic SYNTAX I/II scoring system. Risk stratification of the SYNTAX score was defined based on former trials (≤22, 23–32, ≥33). Based on the anatomical SS, the risk stratification of the SYNTAX II score was defined based on the statistical tertial results according to the work of Kang *et al*. [17]. The population was divided into three strata according to the SSII: low SSII (SSII ≤28), 46 patients (28.75%); intermediate SSII (30 < SSII < 40), 88 patients (55.00%); high SSII (SSII ≥40), 26 patients (16.25%). Two interventional doctors logged on to the official website and independently calculated scores based on images from coronary angiography images. If they disagreed on a patient, a third doctor would decide if the controversial lesion should be included.

### 2.3 Measurements of QFR

In this study, all patients were eligible for QFR measurements. QFR was performed for all lesions calculated in the SS before and after PCI. An offline QFR analysis was performed by trained technicians of our department using the QFR system (AngioPlus, Pulse Medical Imaging Technology, Shanghai, China). All data were calculated by two cardiologists who passed the training test independently. The cut-off value for the physiological significance of QFR was 0.85 [18].

### 2.4 MACEs and Follow-up

MACEs were defined as the composite of cardiac deaths, any myocardial infarction, or ischemia-driven revascularization. An independent and blinded clinical event committee adjudicated all adverse events. A cardiologist followed patients in the clinic at the end of the study period. Telephone or WeChat were used to contact patients once a month.

### 2.5 Statistical Analysis

Continuous variables were expressed as the mean ± SD and compared using one-way analysis of variance (ANOVA). Categorical variables were presented as frequencies and percentages and compared using the chi-square or Fisher’s exact tests. Kaplan–Meier analysis compared time to MACEs according to risk stratification of SSI/II and functional SYNTAX score (FSS) I/II, with significance assessed using the log-rank test for trend. A univariate Cox regression analysis was used to identify independent predictors of MACEs during follow-up. A receiver operating characteristic (ROC) curve was plotted to compare SSI/II and FSSI/II prediction capabilities for MACEs. The area under the curve was compared using the DeLong method. A value of *p* < 0.05 was considered statistically significant. All statistical analyses were performed using SPSS 27.0 software (SPSS Inc, Chicago, IL, USA).

## 3. Results

### 3.1 Population and Risk Stratification

**Fig. 1.**
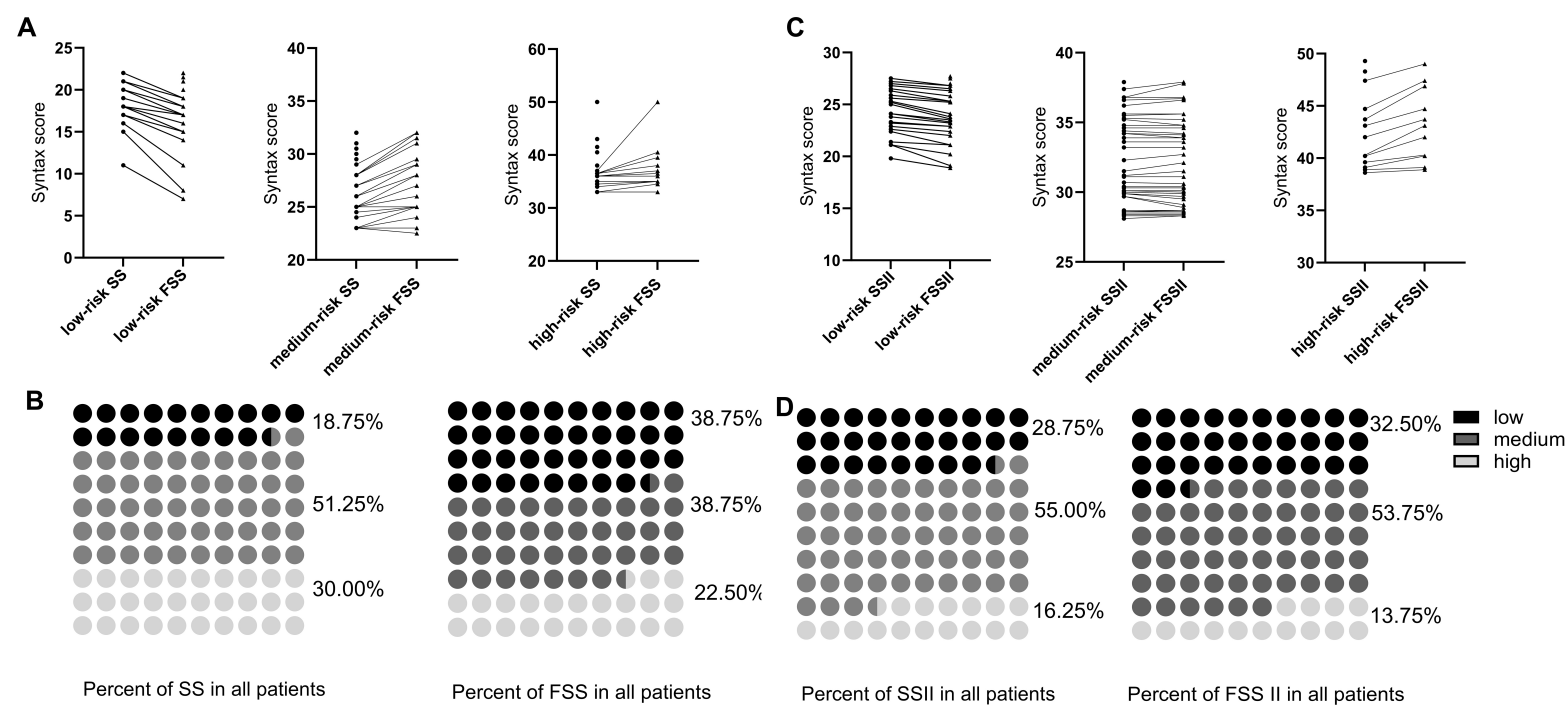
**Risk stratification of patients**. (A) Regroup of patients in different risk stratification after recalculation by FSS. (B) Percentage change of different risk stratification after recalculation by FSS. (C) Regroup of patients in different risk stratification after recalculation by FSSII. (D) Percentage change of different risk stratification after recalculation by FSSII. SS, SYNTAX score; FSS, functional SYNTAX score; SSII, SYNTAX score II; FSSII, functional SYNTAX score II.

A total of 160 patients with multivessel CAD were enrolled and completed the follow-up. All patients underwent complete revascularization. Sirolimus-eluting stents were used in all patients in this study, and no bare metal stents were used. The baseline characteristics of the study population were grouped by risk stratification based on SSI and SSII, shown in Tables 1,2. After recalculation of SS, 7.5% of patients (N = 12) were reclassified from the high-risk group into the low-risk group, and 12.5% (N = 20) were reclassified from the medium group into the low-risk group (Table 1, Fig. 1A,B). The cut-off value for SSII was defined as ≤28, 28–38, and >38 after ROC analysis. Results also showed that 2.5% of patients (N = 4) were reclassified from the high-risk group into the low-risk group, and 1.25% (N = 2) were reclassified from the medium group into the low-risk group (Table 2, Fig. 1C,D).

**Table 1.** **Patients’ baseline characteristics based on SS and FSS risk stratification**.

Variables		All patients	SS				FSS			
			Low risk	Medium risk	High risk	*p*-value	Low risk	Medium risk	High risk	*p*-value
		N = 160	(N = 30)	(N = 82)	(N = 48)		(N = 62)	(N = 62)	(N = 36)	
Age, years		67.15 ± 9.64	63.87 ± 6.65	69.44 ± 9.49	65.29 ± 10.61	0.006	65.87 ± 8.99	69.55 ± 9.21	65.22 ± 10.80	0.040
Male		128 (80.00)	24 (80.00)	64 (78.05)	40 (83.33)	0.768	12 (19.35)	14 (22.58)	6 (16.67)	0.769
Hypertension		116 (72.50)	18 (40.00)	66 (80.49)	32 (66.67)	0.055	48 (77.42)	42 (67.74)	26 (72.22)	0.484
Diabetes		66 (41.25)	12 (6.67)	34 (41.46)	20 (41.67)	0.988	28 (45.16)	22 (35.48)	16 (44.44)	0.498
Hypercholesterolemia		14 (8.75)	2 (6.67)	6 (7.32)	6 (12.50)	0.653	4 (6.45)	6 (9.68)	4 (11.11)	0.771
Previous MI		4 (2.50)	0 (0.00)	2 (2.44)	2 (4.17)	0.656	0 (0.00)	4 (6.45)	0 (0.00)	0.078
LVEF		61.00 ± 5.76	62.20 ± 2.35	60.41 ± 6.79	61.25 ± 5.29	0.128	60.52 ± 5.81	61.84 ± 5.76	60.39 ± 5.68	0.343
eGFR		83.41 ± 21.77	84.69 ± 14.01	80.19 ± 21.28	88.09 ± 25.70	0.329	85.71 ± 16.29	77.60 ± 24.07	89.44 ± 23.98	0.019
Medications										
	Antiplatelet	160 (100)	30 (100.00)	82 (100.00)	48 (100.00)	-	62 (100.00)	62 (100.00)	36 (100.00)	-
	Stains	160 (100)	30 (100.00)	82 (100.00)	48 (100.00)	-	62 (100.00)	62 (100.00)	36 (100.00)	-
	ACEI/ARB	116 (72.50)	18 (60.00)	64 (78.05)	34 (70.83)	0.159	40 (64.52)	46 (74.19)	30 (83.33)	0.123
	β-blockers	112 (70.00)	14 (46.67)	60 (73.17)	38 (79.17)	0.006	38 (61.29)	46 (74.19)	28 (77.78)	0.149
Angiographic										
	SYNTAX score	28.26 ± 7.19	18.07 ± 2.72	26.96 ± 2.68	36.83 ± 3.73	<0.001	22.09 ± 4.65	29.57 ± 4.75	36.61 ± 4.04	<0.001
	Bifurcation lesion	6 (3.75)	0 (0.00)	4 (4.88)	2 (4.17)	0.264	2 (3.23)	2 (3.23)	2 (5.56)	0.757
	Total occlusion	56 (35.00)	6 (20.00)	30 (36.59)	20 (41.67)	0.136	16 (25.81)	22 (35.48)	18 (50.00)	0.053
	Severe tortuosity	2 (1.25)	0 (0.00)	0 (0.00)	2 (4.17)	0.123	0 (0.00)	0 (0.00)	2 (5.56)	0.050
	Severe calcification	8 (5.00)	2 (6.67)	4 (4.88)	2 (4.17)	0.795	6 (9.68)	0 (0.00)	2 (5.56)	0.033

SS, SYNTAX score; FSS, functional SYNTAX score; MI, myocardial infarction; eGFR, estimated glomerular filtration rate; LVEF, left ventricular ejection fraction; ACEI, angiotensin-converting enzyme inhibitor; ARB, angiotensin receptor blocker. Values are the mean ± SD, or n (%).

**Table 2.** **Patients’ baseline characteristics based on SSII and FSSII risk stratification**.

Variables		SSII				FSSII			
		Low risk	Medium risk	High risk	*p*-value	Low risk	Medium risk	High risk	*p*-value
		(N = 46)	(N = 88)	(N = 26)		(N = 52)	(N = 86)	(N = 22)	
Age, years		58.39 ± 6.93	69.09 ± 7.42	76.08 ± 8.72	<0.001	60.08 ± 8.21	69.35 ± 7.99	75.27 ± 8.31	<0.001
Male		42 (91.30)	70 (79.55)	16 (61.54)	0.009	48 (92.31)	60 (69.77)	14 (63.64)	0.003
Hypertension		32 (69.57)	64 (72.73)	20 (76.92)	0.796	38 (73.08)	60 (69.77)	18 (81.82)	0.525
Diabetes		18 (39.13)	36 (40.91)	12 (46.15)	0.841	20 (38.46)	36 (41.86)	10 (45.45)	0.843
Hypercholesterolemia		4 (8.70)	8 (9.09)	2 (7.69)	1.000	4 (7.69)	8 (9.30)	2 (9.09)	1.000
Previous MI		0 (0.00)	4 (4.55)	0 (0.00)	0.285	0 (0.00)	4 (4.65)	0 (0.00)	0.273
LVEF		61.61 ± 4.69	60.89 ± 6.06	60.31 ± 6.52	0.633	61.65 ± 4.47	60.35 ± 6.87	62.00 ± 2.82	0.298
eGFR		95.59 ± 18.59	83.89 ± 19.16	60.89 ± 6.06	<0.001	94.39 ± 17.86	83.50 ± 19.53	57.10 ± 15.75	<0.001
Medications									
	Antiplatelet	46 (100.00)	88 (100.00)	26 (100.00)	-	52 (100.00)	86 (100.00)	22 (100.00)	-
	Stains	46 (100.00)	88 (100.00)	26 (100.00)	-	52 (100.00)	86 (100.00)	22 (100.00)	-
	ACEI/ARB	28 (60.87)	64 (72.73)	24 (92.31)	0.016	34 (65.38)	62 (72.09)	20 (90.91)	0.079
	β-blockers	30 (65.22)	64 (72.73)	18 (69.23)	0.664	34 (65.38)	64 (74.42)	14 (63.64)	0.417
Angiographic									
	SYNTAX score	26.04 ± 8.86	28.78 ± 6.64	30.39 ± 4.44	0.028	26.12 ± 8.37	28.74 ± 6.72	31.41 ± 4.04	0.009
	Bifurcation lesion	2 (4.35)	4 (4.55)	0 (0.00)	0.729	2 (3.85)	4 (4.65)	0 (0.00)	0.859
	Total occlusion	14 (30.43)	36 (40.91)	6 (23.08)	0.183	16 (30.77)	34 (39.53)	6 (27.27)	0.414
	Severe tortuosity	0 (0.00)	2 (2.27)	0 (0.00)	0.682	0 (0.00)	2 (2.33)	0 (0.00)	0.648
	Severe calcification	0 (0.00)	4 (4.55)	4 (15.38)	0.013	2 (3.85)	4 (4.65)	2 (87.50)	0.684

SSII, SYNTAX score II; FSSII, functional SYNTAX score II; MI, myocardial infarction; eGFR, estimated glomerular filtration rate; LVEF, left ventricular ejection fraction; ACEI, angiotensin-converting enzyme inhibitor; ARB, angiotensin receptor blocker. Values are the mean ± SD, or n (%).

### 3.2 Univariable Analysis of MACEs in All Patients

Thirty-four patients suffered MACEs. An univariable analysis was performed on clinical and anatomy factors in all patients for each MACE. The results showed only left ventricular ejection fraction (LVEF) significantly influenced MACEs (Table 3). Except for SS, FSS, and the residual SYNTAX score (rSS) system, the other five scoring systems showed significant predictive ability for MACEs. SSII (HR: 1.072, 95% CI: 1.026–1.120, *p* = 0.002) and functional SYNTAX score II (FSSII) (HR: 1.069, 95% CI: 1.025–1.115, *p* = 0.002) showed a better correlation with MACEs than the other scoring system (Table 4).

**Table 3.** **Predictors of MACEs in all patients according to univariable analysis**.

Variables	HR (95% CI)	*p*-value
Age, years	1.035 (0.999–1.072)	0.055
Male	1.252 (0.602–2.605)	0.547
Hypertension	1.881 (0.782–4.523)	0.158
Diabetes	1.372 (0.721–2.608)	0.335
Hypercholesterolemia	1.205 (0.425–3.416)	0.726
eGFR	1.001 (0.985–1.017)	0.941
LVEF	1.104 (1.003–1.216)	0.043
Bifurcation lesion	1.127 (0.268–4.733)	0.871
Total occlusion	0.990 (0.474–2.067)	0.979
Severe tortuosity	0.048 (0.000–715.463)	0.535
Severe calcification	2.124 (0.498–9.069)	0.309

MACEs, major adverse cardiovascular events; eGFR, estimated glomerular filtration rate; LVEF, left ventricular ejection fraction; HR, hazard ratio; CI, confidence interval.

**Table 4.** **Univariable analysis of SSI/II and QFR-SSI/II on MACEs in all patients**.

Variables	HR (95% CI)		*p*-value
SS	1.048 (0.999–1.099)	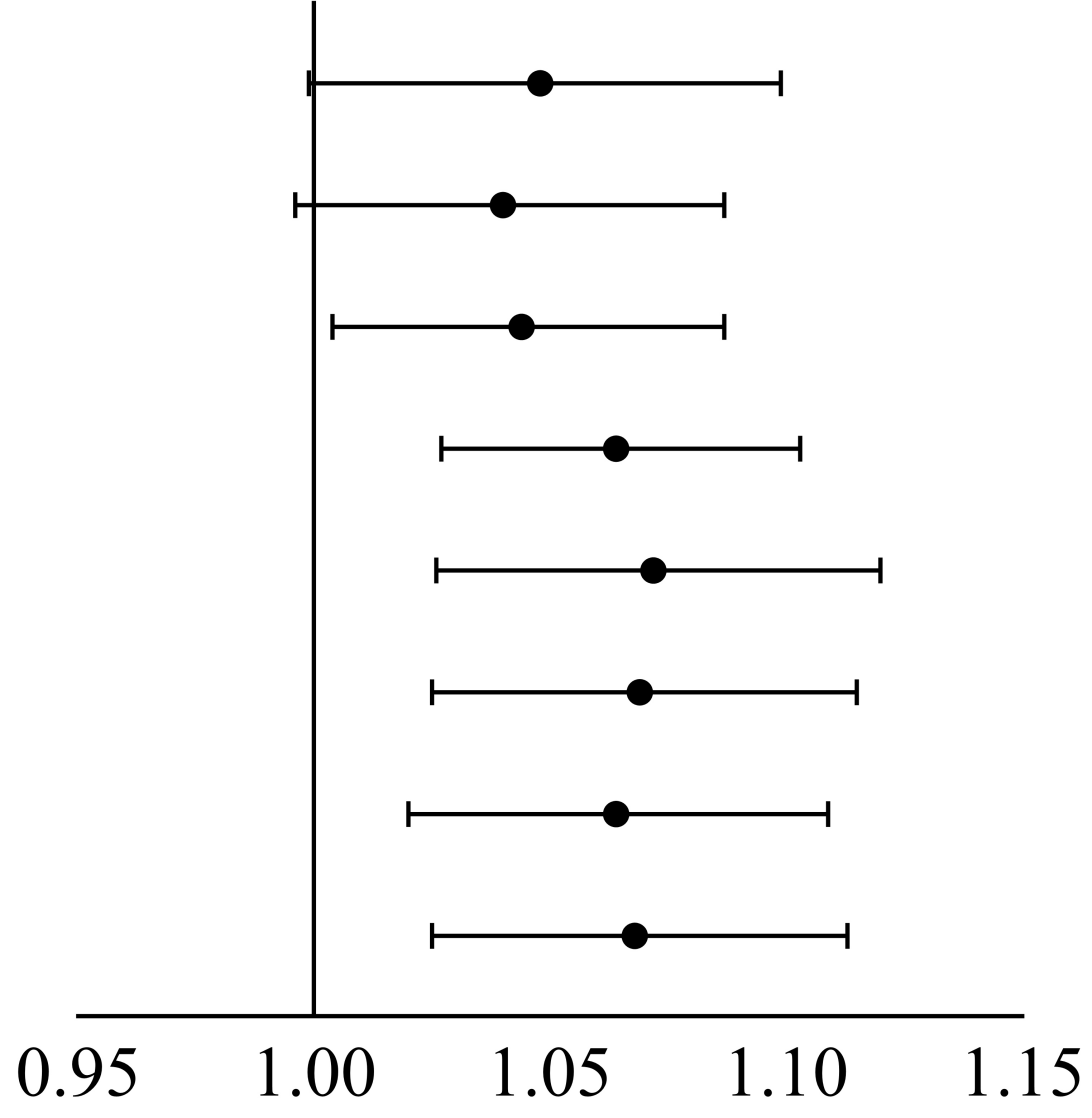	0.056
FSS	1.040 (0.996–1.087)		0.077
rSS	1.044 (1.004–1.087)		0.032
rFSS	1.064 (1.027–1.103)		<0.001
SSII	1.072 (1.026–1.120)		0.002
FSSII	1.069 (1.025–1.115)		0.002
rSSII	1.064 (1.020–1.109)		0.004
rFSSII	1.068 (1.025–1.113)		0.002

rSS, residual SYNTAX score; rFSS, residual SYNTAX score; MACEs, major adverse cardiovascular events; QFR, quantitative flow ratio; HR, hazard ratio; CI, confidence interval; SS, SYNTAX score; FSS, functional SYNTAX score.

### 3.3 Prognostic Value of Different Score Systems

**Fig. 2.**
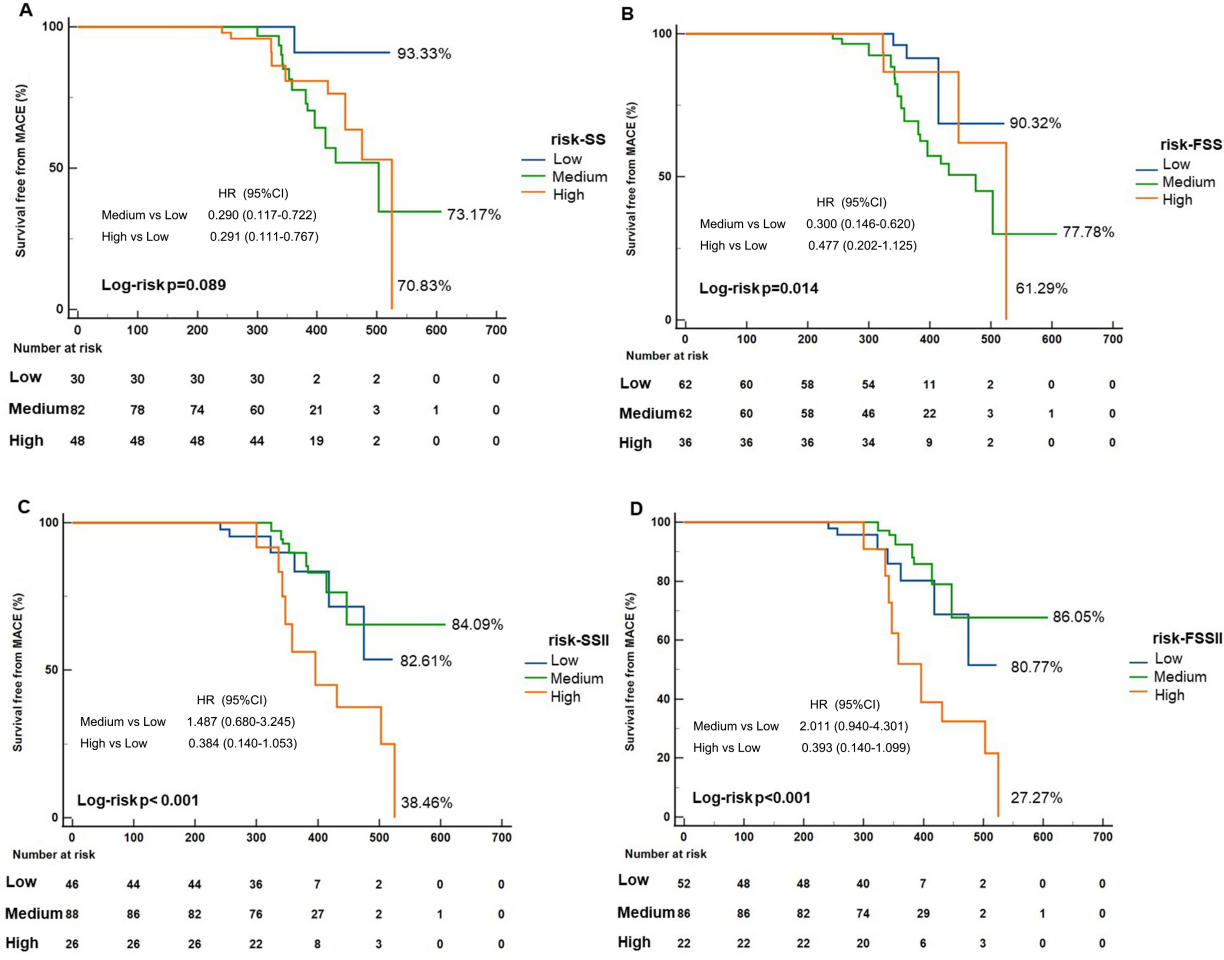
**Survival free from MACEs in different risk stratification under different scoring systems**. (A,B) After recalculation by QFR-based SS, the survival-free ratio decreased from 70.83% to 61.29% in the high-risk group. (C,D) After recalculation by QFR-based SSII, the survival-free ratio decreased from 38.46% to 27.27% in a high-risk group and increased from 84.09% to 86.05% in a low-risk group. SS, SYNTAX score; FSS, functional SYNTAX score; SSII, SYNTAX score II; FSSII, functional SYNTAX score II; QFR, quantitative flow ratio; MACEs, major adverse cardiovascular events; HR, hazard ratio; CI, confidence interval.

Log-risk analysis showed significant differences from different risk stratification on MACEs except SS (log-risk *p* = 0.089). After recalculating the SS, the survival-free ratio stratified by FSS decreased from 70.83% to 61.29% in thehigh-risk group. After recalculating the SSII, the survival-free ratio stratified by FSSII decreased from 38.46% to 27.27% in the high-risk group and increased from 84.09% to 86.05% in the low-risk group (Fig. 2).

**Fig. 3.**
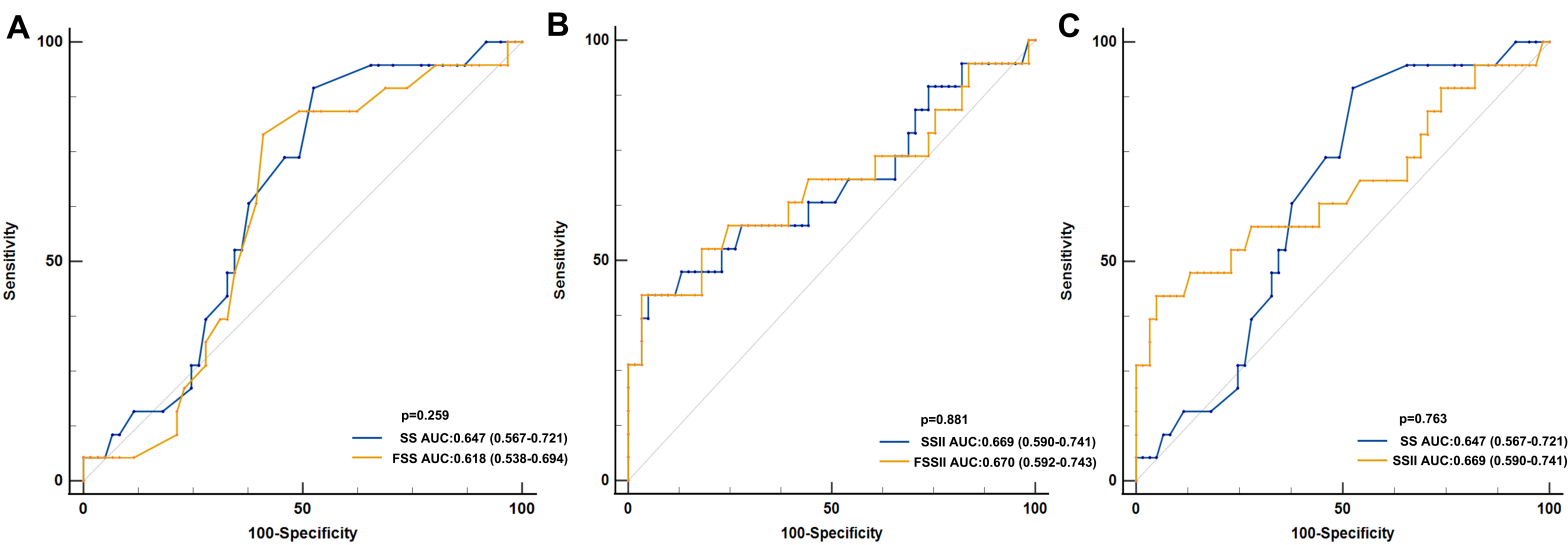
**Receiver-operator characteristic (ROC) curve analysis for different scoring systems**. (A) Classified by SS and FSS. (B) Classified by SSII and FSSII. (C) Classified by SS and SSII. AUC, area under the curve; SS, SYNTAX score; FSS, functional SYNTAX score; SSII, SYNTAX score II; FSSII, functional SYNTAX score II.

Although the area under the curve (AUC) of SSII (AUC = 0.669) and FSSII (AUC = 0.670) were larger than the SS (AUC = 0.647) and FSS (AUC = 0.618), no significant differences were found. Furthermore, FSSII showed no superior predictive ability than SSII. There were also no remarkable differences in predictive ability between the SS and SSII groups (Fig. 3).

## 4. Discussion

The main finding of this study was that either SS or SSII combined with the functional score system could decrease the portion of high-risk and medium-risk patients. Furthermore, the study also showed that SSII and FSSII had accurate predictive abilities. Risk stratification based on FSSII showed a better MACE predictive ability. These findings revealed that a non-invasive QFR-based SYNTAX score II might be a good prognostic predictor in multivessel disease patients with different risk stratification.

**Fig. 4.**
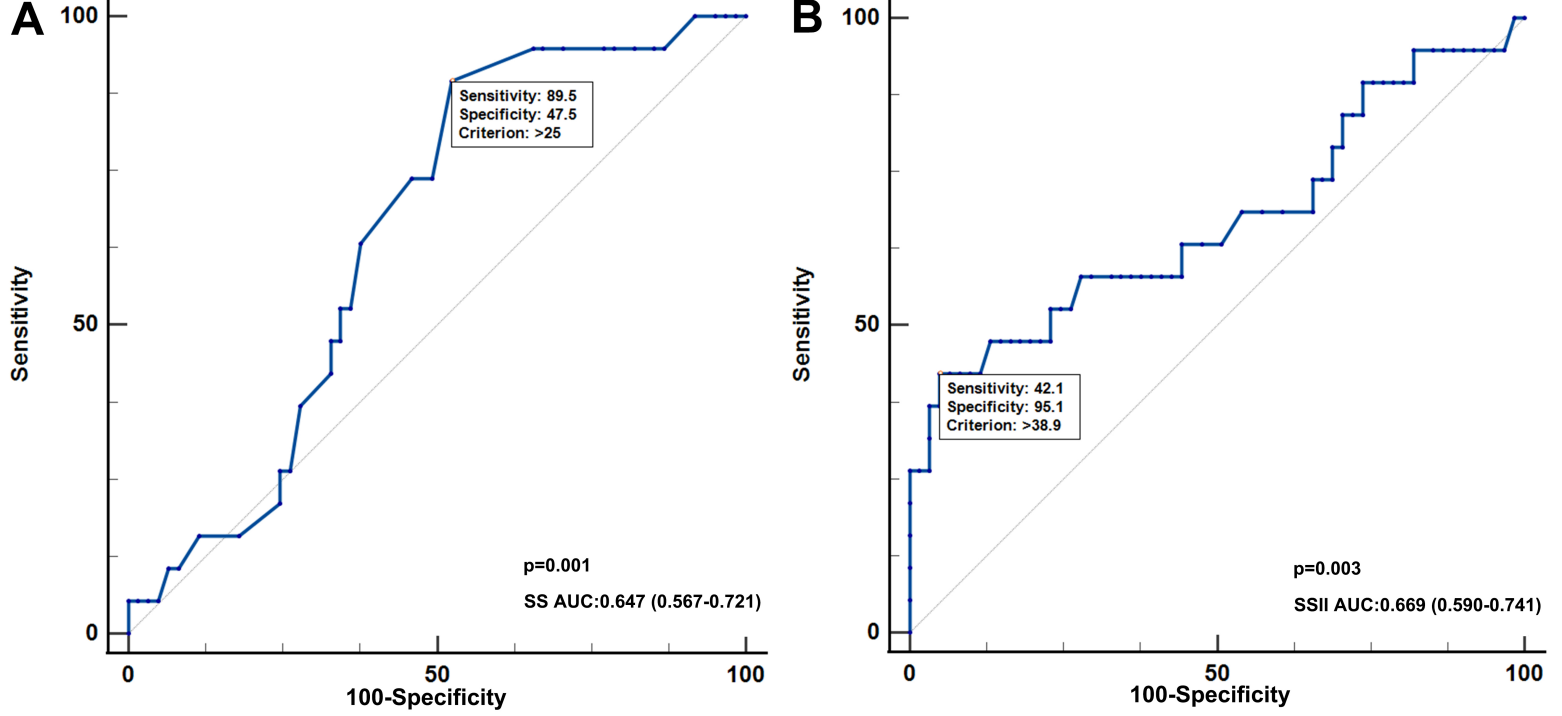
**Receiver-operator characteristic (ROC) curve analysis of SS and SSII for MACE patients**. (A) The predictive ability of SS in diagnosis of MACE in all patients. (B) The predictive ability of SSII in diagnosis of MACEs in all patients. SSII, SYNTAX score II; AUC, area under the curve; SS, SYNTAX score; MACE, major adverse cardiovascular event.

SYNTAX score was a classical anatomy score system to predict the prognosis of multivessel disease patients. In high-risk SS multivessel disease patients, coronary artery bypass surgery (CABG) showed a mortality benefit over PCI [19, 20]. SYNTAX score II decreased the bias of clinical factors by bringing the factors of age, creatinine clearance (CrCl), LVEF, sex, left main lesion, chronic obstructive pulmonary disease (COPD), and peripheral vascular disease (PVD) into the calculation. It even showed a better predictive ability than SS in PCI patients with complex coronary disease [2, 21]. In this study, we calculated SSII cut-off values of 28, 28–38, and >38 based on the ROC analysis (AUC = 0.669, 95% CI: 0.590–0.741, *p* = 0.033, Fig. 4). Using this criterion, high-, medium- and low-risk groups showed significant differences in the survival-free ratio from MACEs (log-rank risk, *p* < 0.001). Considering the unclear cut-off value of SSII in most studies, 28, 28–38, >38 were used for risk stratification in the SSII criterion.

Nam *et al*. [11] integrated the SS and FFR to develop the concept of the functional SYNTAX score. They calculated the SS only in vessels with low FFR (≤0.8) and showed that the FSS better predicted clinical outcomes than SS. Subsequently, 38% of patients in the high-risk SS tertile were moved to the medium- or lowest-risk FSS groups, whereas 59% of the medium-risk SS tertile moved to the lowest-risk FSS group [11]. In this study, we sought to prove the ability of non-invasive functional integrated scores such as the SS or SSII to predict MACEs. The accuracy of QFR has been shown to better identify hemodynamically significant coronary stenosis compared with FFR [13]. In a study by Smit *et al*. [18], 181 (54.2%) coronary arteries had a QFR value ≥0.86, and only 9 (5.0%) of these coronary arteries had an invasive FFR of ≤0.80. Based on the cut-off value of 0.85, functional SS and SSII were used to reclassify the risk stratification groups of patients in our study. The results showed that high-risk patients were resigned into medium- and low-risk patients after either SS or SSII were corrected by QFR (Fig. 1). There was no significant difference in the different groups in SS score; however, FSS, SSII, and FSSII showed marked differences in these corresponding groups. Only 4 (2.5%) patients were reassigned from the high-risk group into the medium-risk group, guided by FSSII (Fig. 1).

Studies on the value of FFR after PCI when predicting the prognosis of patients are relatively common [11], but there are still few studies on QFR after PCI [16]. In the study by Xu* et al*. [22], a QFR-guided PCI strategy improved 1-year clinical outcomes of patients compared with standard angiography guidance. We analyzed the Kaplan–Meier curves in different groups under different scoring systems to evaluate the prognostic value of the different score systems. We found that after recalculation using FSS, the survival free ratio from MACEs in different risk stratifications showed significant differences in high-, medium- and low-risk groups compared with SS (log-risk *p* = 0.089 in SS, log-risk *p* = 0.014 in FSS). In Table 3, the univariable analysis showed no significant impact on MACEs when clinical factors were independently assessed, except LVEF. However, when clinical factors were integrated into SS for SSII, either SSII (HR: 1.072, 95% CI: 1.026–1.120, *p* = 0.002) or FSSII (HR: 1.069, 95% CI: 1.025–1.115, *p* = 0.002) showed significant effects on MACEs. In addition, based on FSSII risk stratification, survival-free from the MACE ratio decreased from 38.48% to 27.27% in the high-risk group and increased from 84.09% to 86.05% in the low-risk group compared with SSII risk stratification. Considering these results, FSSII may be an appropriate scoring system to predict MACEs in patients with different risk stratification.

Unfortunately, FSSII showed better AUC in predicting MACEs, but it did not result in statistically significant results compared with the FSS system (*p* = 0.881). rSS, rFSS, rSSII, and rFSSII were also analyzed in this study. Even though they were independent predictors of MACEs, they did not meet statistical significance when compared. In recent studies, 0.80 was used for the cut-off value to decide the physiological function of lesions [22, 23]. A lower cut-off value will change the number of patients in different risk stratification based on the FSSII system. Considering the cut-off value and sample size, a further study based on the results of this study may enhance the predictive ability of FSSII.

## 5. Limitation

This study’s limitation was its small sample size, which lowered its statistical efficiency. Furthermore, some studies showed that 0.80 could be a possible cut-off value for QFR. Therefore, the different criteria of QFR may also affect the efficacy of FSSII in predicting MACEs. Moreover, the discrepancy between different methods of calculating QFR was also a factor that may affect the result. Considering these limitations, our ongoing prospective study with a bigger sample may provide more definitive results in the near future.

## 6. Conclusions

Compared with classical SS and SSII, FSS and FSSII provided better risk stratification criteria to predict the occurrence of MACEs by regrouping patients. SSII and FSSII might be good predictors for MACEs in multivessel patients.

## Availability of Data and Materials

The original contributions presented in the study are included in the article, further inquiries can be directed to the corresponding author.
